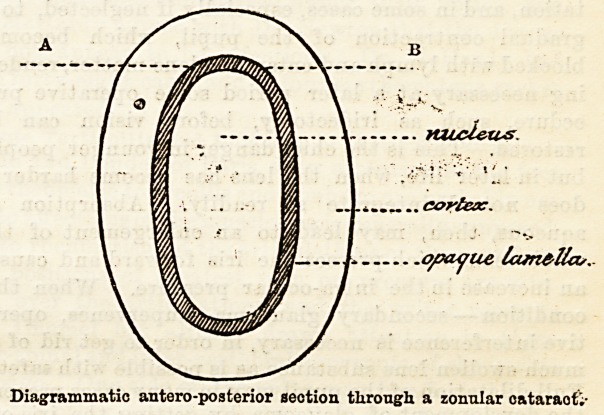# Cataract as It Is Met with in Ordinary Practice

**Published:** 1895-05-18

**Authors:** R. Lawford Knaggs

**Affiliations:** Assistant Surgeon to the Leeds General Infirmary, and Ophthalmic Surgeon to the Leeds Public Dispensary


### Medical Progress and Hospital Clinics.

[*The Editor will be glad to receive offers of co-operation and contributions from members of the profession. All letters should be addressed to* The Editor, The Lodge, Porchester Square, London, W.]

### CATARACT AS IT IS MET WITH IN ORDINARY PRACTICE.

By R. Lawford Knaggs, Assistant Surgeon to the Leeds General Infirmary, and Ophthalmic Surgeon to the Leeds Public Dispensary.

Of the various conditions regarded as the peculiar property of "the specialist," cataract is one of such frequent occurrence in ordinary practice either as a congenital or as an acquired condition, as a result of injury, or as an accompaniment of other disease, that it possesses an interest which all can appreciate and to some extent share.

In advanced cases the presence of a cataract is plain enough, even though the pupil is small, but in the earlier stages and when the patient first begins to complain of failing sight, it may be impossible to detect the opacities in the lens unless the pupil is specially dilated.

Dilatation is usually produced by the use of atropine (liq. atrop. sulph.), or homatropine (hydrobromate of homatropine gr. iv. ad. ℥i.), drops or discs, but as the inconvenience produced by these drugs upon the accommodation is well known and often prevents their employment, it is worth remembering that a 2 per cent. or 4 per cent. solution of cocaine will answer sufficiently well for practical purposes, besides being more free from the risk of inducing glaucoma.

The recognition of cataract presents no difficulty, if the gas light is focussed sideways upon the dilated pupil with a convex lens, but occasionally when this oblique illumination fails to show any pathological alterations, early lens changes may be demonstrated by the ophthalmoscope as dark striæ against the red fundus reflex or occasionally as lines closely resembling flaws in glass.

On the other hand there are conditions which with oblique illumination appear to simulate cataract, especially to the inexperienced, but when the fundus is lit up with reflected light the lens is seen to be perfectly transparent. Consequently these two methods of examination (oblique and direct) should always be employed together—the one to check or confirm the other.

At some period of his career every practitioner will come in contact with three widely different varieties of cataract; (1) the commonest form, in which the nucleus or the cortex is first affected, and the changes tend to involve the whole lens. We need not here concern ourselves with the names that are given to the different varieties of this group. (2) Lamellar or zonular cataract, in which the opacity is only partial, the superficial lamellæ and the nucleus being transparent but a shell of opacity composed of several degenerated lamellæ separating the nucleus from the cortex. (3) Traumatic cataract, the most urgent group of all.

The recognition of the first variety is too simple for further comment, because the failure of sight in those who have been accustomed to see well, if not relieved by glasses, should lead the medical man to examine the eyes; and inability to realise the true condition is impossible if the investigation is properly made.

But with zonular cataract it is different. In consequence of the thinness of the opaque lamella a certain amount of vision is possible, and as the children (for this condition, when present, is usually first detected in early life) have probably suffered from it from infancy, the defective sight may not be noticed till school work is commenced, and then only after a fruitless search for glasses do they reach the doctor's hands. For the diagnosis a dilated pupil is essential. Then oblique illumination shows a circular grey opacity not extending to the edge of the lens and situated at a varying depth in its substance. Possibly the opacity may be seen to consist of a superficial and a deep layer. By direct illumination with the ophthalmoscope a dark grey disc transmitting a greater or less amount of reflected light is found to occupy the centre of the pupil. This disc is surrounded by a ring of well marked red reflex, whilst its margin is darker than the central portion because there is more degenerated

Diagrammatic antero-posterior section through a zonular cataract.

lens tissue for the light to traverse at the edge of the lamella, where the anterior layer bends round to join the posterior. (*Vide* diagram A B.)

The diagnosis of traumatic cataract presents no difficulty. The history of injury, the frequent but not universal presence of a penetrating wound of the eye—most frequently in the cornea—the rapid development of a white opacity in the lens, and possibly some opaque lens matter blocking the pupil or occupying the anterior chamber, are facts of so much importance and so obvious as to attract attention at once. But if this condition is more readily recognised, or less likely to be overlooked in the mass of work which engages the general practitioner's attention, it is one which requires on his part a certain ready knowledge of treatment.

The other varieties of cataract, when recognised, he will probably prefer to hand over to the ophthalmic surgeon, but in the latter treatment is called for at once, and, if promptly employed, it is possible in many instances to conduct the case almost completely to a successful issue.

It is a remarkable fact that though many cases of traumatic cataract result from penetration of the eyeball by an instrument that is anything but clean—as, for instance, by the prong of a fork slipping when being used to unpick a knot in a bootlace—yet septic inflammation is so rare that, in the large majority of instances, no anxiety need be felt upon this score.

As a result of the wound in the capsule the lens matter absorbs aqueous, swells up, becomes opaque, and eventually dissolves, the whole lens being removed in the course of a few months.

The surgeon's object is to allow this absorption to go on, and he takes measures to keep up conditions favourable to it, and to prevent complications. A solution of sulphate of atropine (four grains to the ounce) is dropped into the eye several times a day to ensure full dilatation of the pupil, so that the softened lens matter may project or drop into the anterior chamber and be exposed to the solvent action of the aqueous humour.

If the pupil can be kept well dilated iritis may be prevented; and iritis produced by the pressure of the irregular masses of swollen lens substance upon the posterior surface of the iris is one of the two most important complications usually met with in traumatic cataract. It leads to adhesions of the iris to the lens substance, to a difficulty in keeping up the dilatation, and in some cases, especially if neglected, to a gradual contraction of the pupil, which becomes blocked with lymph and entangled lens matter, rendering necessary at a later period some operative procedure, such as iridectomy, before vision can be restored. This is the chief danger in younger people, but in later life, when the lens has become harder it does not disintegrate so readily. Absorption of aqueous, then, may lead to an enlargement of the whole lens, which presses the iris forward and causes an increase in the intra-ocular pressure. When this condition — secondary glaucoma—supervenes, operative interference is necessary, in order to get rid of as much swollen lens substance as is possible with safety. Full dilatation of the pupil may in many cases prevent the development of glaucoma by getting the iris out of the way of the lens, but in others, from the nature of circumstances, this complication may be unavoidable, and when it does occur it calls for immediate action if the eye is to be saved.

## Figures and Tables

**Figure f1:**